# The Institutionalisation of Brazilian Older Abused Adults: A Qualitative Study among Victims and Formal Carers

**DOI:** 10.3390/geriatrics8030065

**Published:** 2023-06-06

**Authors:** Dayane Ribeiro, Lígia Carreira, Maria Aparecida Salci, Francielle Renata Danielli Martins Marques, Adriana Gallo, Wanessa Baccon, Vanessa Baldissera, Carlos Laranjeira

**Affiliations:** 1 Postgraduate Nursing Department, State University of Maringá, Avenida Colombo, 5790—Campus Universitário, Maringá 87020-900, PR, Brazil; dayaneakinara@hotmail.com (D.R.); ligiacarreira.uem@gmail.com (L.C.); masalci@uem.br (M.A.S.); franrenata.martins@gmail.com (F.R.D.M.M.); adrianagallo.particular@gmail.com (A.G.); wanessabaccon@hotmail.com (W.B.); vdabaldissera2@uem.br (V.B.); 2 School of Health Sciences, Polytechnic University of Leiria, Campus 2, Morro do Lena, Alto do Vieiro, Apartado 4137, 2411-901 Leiria, Portugal; 3 Centre for Innovative Care and Health Technology (ciTechCare), Polytechnic University of Leiria, Campus 5, Rua de Santo André-66–68, 2410-541 Leiria, Portugal; 4 Comprehensive Health Research Centre (CHRC), University of Évora, 7000-801 Évora, Portugal

**Keywords:** aged, elder abuse, institutionalisation, neglect, frailty, Brazil

## Abstract

Abuse against elders is acknowledged as a severe and pervasive problem in society. If support services are not tailored to the victims’ knowledge or perceived needs, the intervention is likely to be unsuccessful. This study aimed to explore the experience of institutionalisation of abused older people from the perspective of the victims and their formal carers in a Brazilian social shelter. A qualitative descriptive study was performed with 18 participants, including formal carers and older abused persons admitted to a long-term care institution in the south of Brazil. Qualitative thematic analysis was used to analyse the transcripts of semi-structured qualitative interviews. Three themes were identified: (1) personal, relational, and social bonds: broken or weakened; (2) denial of the violence suffered; and (3) from imposed protection to compassionate care. Our findings provide insights for effective prevention and intervention measures in elder abuse. From a socio-ecological standpoint, vulnerability and abuse might be averted at the community and societal levels (e.g., education and awareness of elder abuse) by creating a minimum standard for the care of older individuals (e.g., law or economic incentives). Further study is needed to facilitate recognition and raise awareness among individuals in need and those offering assistance and support.

## 1. Introduction

Population ageing is an irreversible global trend. This phenomenon is the result of increasing average life expectancy, even in countries with relatively young populations [1]. Currently, families are becoming smaller and include a greater number of older relatives, often with different generations living under the same roof.

In Brazil, there were about 14 million older people (≥60 years old) in 2000. This population reached 29 million in 2020 and is projected to exceed 35 million by 2025 [2,3]. Globally, the number of people aged 65 and over is estimated to double from 761 million in 2021 to 1.6 billion by 2050, and people aged 80 and over are increasing even faster [1].

Concurrently, abuse against older people—considered a public health problem by the World Health Organization (WHO)—has been increasing in the healthcare and general care networks, reaching all social classes and becoming a reality in the life of this age group [4,5].

There is a paucity of violence statistics against older people [6]. In Brazil, studies have documented a prevalence of violence against the elderly ranging from 13% to 14.4% [7,8,9]. These values are higher than those found in Europe (5.6%), North America (8.1%), and others published by the WHO (4 to 6%), but are lower than rates found in African (43.7%) and Asian (49.1%) countries [10]. In Brazilian legislation, elder abuse requires mandatory notification, but is underreported, which undermines epidemiological data, consolidates the phenomenon’s invisibility, and hinders preventive actions [4,10,11,12]. Elder abuse is a culturally sensitive issue [13] and can assume various forms, depending on cultural background, values, beliefs, and other complex factors.

In general, elder abuse is defined as the deliberate action or inaction toward an older adult with the intent of causing harm, suffering, deprivation, or death [3,5,6,7,8,9,10]. There are five categories of abuse: “physical abuse, psychological abuse, sexual abuse, economic abuse, and neglect” [10]. A meta-analysis of worldwide studies showed that global elder abuse prevalence in community settings “varied between 11.6% for psychological abuse, 6.8% for financial abuse, 4.2% for neglect, 2.6% for physical abuse, and 0.9% for sexual abuse” [14] (p.e147). Rates of elder abuse are high and have increased during the COVID-19 pandemic [12]. This is not restricted to the family environment, it is also associated with long-stay institutions and community settings [10,15,16].

Empirical evidence on elder abuse in domestic environments shows that family members and close friends are the most frequent perpetrators of abuse, damaging the well-being and physical and psychological health of older adults [5,10,17]. The burden of caring for older individuals falls largely on their families. Traditional cultural ideals of “filial piety” require adult offspring to care for their older parents. The family is the primary context in which human development takes place [18] and when family ties and care possibilities are weakened, the elderly may be exposed to situations of risk, violence [5], and poly-victimisation. Teaster [19] defined elder poly-victimisation as “multiple co-occurring or sequential types of elder abuse by one or more perpetrators or when an older adult experiences one form of abuse perpetrated by multiple others with whom the older adult has a personal, professional, or care recipient relationship in which there is a societal expectation of trust” (p. 290).

In Brazil, the protection and care of older abused victims occur in Long Stay Institutions for the Elderly (LSIEs) organised by the Unified Social Assistance System (SUAS) and operationalised by the Organic Law of Social Assistance (LOAS) [20], which assumes a key role in emergency situations. However, restricted institutional regulations and an absence of a person-centred approach affect residents’ self-determination in everyday activities [21]. Few studies have addressed the institutionalisation of victims of abuse and the fragility of their family relationships [22]. This knowledge gap hinders our use of strategies to optimise the health care of this population [20,23].

Most qualitative studies interviewed professionals regarding elder abuse [17,24] and only a few interviewed the victims [4,25]. Therefore, we lack a holistic portrayal of the phenomenon. This calls for a bio-psychosocial approach incorporating health, safety, and social needs, based on the victim’s priorities. Evidence of elder abuse is relatively recent in terms of intrafamily violence, and, therefore, empirical evidence lacks a theoretical framework within which to understand its multiple manifestations. The socio-ecological model inspired by Bronfenbrenner [18] and Teaster [19] has been widely used to understand elder abuse. It reflects abuse at different levels—micro, meso, exo, and macrosystem—and incorporates the perceptions of elder abuse victims, allowing the exploration of adequate prevention and intervention measures [18,26]. The processes of abuse are based on four critical elements: (1) the characteristics of older victims (i.e., gender of victims, race/ethnicity, poor physical/mental health, functional dependence, and low income/socioeconomic status) and the aggressor risk factors (i.e., mental illness, abuser’s dependency on the victim and substance abuse); (2) the relationship between the victim and the perpetrator (i.e., family, friends, and neighbours), which influences the underlying risk factors of the victim–aggressor dynamics and can increase or decrease the probability of abuse; (3) the characteristics of the community and institutional support, which may place the older person at greater risk or protect them (i.e., safety culture, staffing, and formal support); (4) inhibiting or enhancing social factors (i.e., cultural norms, public awareness, and ageism) of abuse [19,27,28,29]. This approach of examining and dealing with abuse highlights the conditions of abuse, allowing for more effective interventions.

Moreover, elder abuse affects the victims, their families, and the wider society. Adverse individual and socioeconomic effects can increase the risk of morbidity, mortality, hospitalisation, and institutionalisation [30]. To our knowledge, no prior research exists in Brazil about how victims and formal carers perceive elder abuse. Therefore, there is scarce information on whether victims’ perceptions and experiences may enhance their care and, if so, how.

Within the framework of elder abuse, our purpose was to explore the experience of institutionalisation of abused older people from the perspective of the victims and their formal carers in a Brazilian social shelter. We hope to offer a comprehensive picture of individual experiences concerning elder abuse, including how mitigation interventions might be fostered.

## 2. Materials and Methods

### 2.1. Study Design

A qualitative study with a descriptive design using thematic content analysis was chosen for this study. The qualitative approach makes it possible to understand real-world issues, valuing the past and emphasising experiences and what they bring to the present [31]. The study followed the guidelines adopted by the Consolidated Criteria for Reporting Qualitative Research (COREQ) [32].

### 2.2. Setting

The study was conducted in a Long Stay Institution for the Elderly (LSIE) intended for the reception of older people who lack the conditions to stay with their family, who have experienced situations of abuse and neglect, who are homeless and abandoned, or whose ties with family members were weakened or severed. This regular LSIE facility can accommodate 18 people (nine women and nine men) and is located in the south of Brazil (Paraná), where an estimated 18.8% of the population is elderly [33].

### 2.3. Participants and Recruitment

In this study, a convenience sampling method was employed. To acquire the most information, sample diversity was maximised by recruiting older adults with varied histories of abuse and formal Long-Term Care (LTC) workers from different backgrounds. LTC workers “are paid staff, typically nurses and personal carers, providing care and/or assistance to people limited in their daily activities at home or in institutions” [34] (p. 1). The eligibility criteria are illustrated in Table 1.

Of the 18 older victims admitted to the unit, two were admitted by court order when younger than 60 years and were not considered eligible for the study. Among the remaining candidates, seven had cognitive impairment [36] and one was admitted to the hospital during the data collection period. The eight eligible seniors were invited to participate and informed about the research objective, the topic under study, and the possible risks of discomfort following the recollection of memories and family and life history.

Of the 28 LTC workers, ten were eligible and invited to participate. 

No invitations were refused and the total number of participants in the research corresponded to 18 people: 8 older victims and 10 formal carers.

### 2.4. Data Collection

Data collection took place between August 2019 and March 2020, a period that preceded the COVID-19 pandemic. Two data collection techniques were used: document analysis and qualitative interview. Initially, personal, social, and institutional information was collected from the individual records of the older victims; subsequently, interviews were conducted using a script of semi-structured questions. This instrument was developed based on the previous literature [13,23] and the authors’ practical knowledge of the research issue. Although the guide was not pilot-tested, questions were rephrased throughout the interviews to increase clarity and understanding. Data collection was conducted by a researcher linked to the researched institution, which allowed a closer relationship with the research participants and an adequate understanding and commitment to the study, indispensable elements throughout the collection process.

All interviews were scheduled according to the participants’ availability and audio-recorded for transcription. The main researcher conducted the interviews in a reserved room at the institution, without interruptions. At the beginning of each interview, the researcher introduced herself and explained the aims of the study. The interviews with older adults were followed by a semi-structured script: “Tell me about your life and care needs before your arrival at the LSIE”; “How has your connection with your family evolved over time?”; “How do you feel about your situation in this institution?”; and “What changes have occurred in your life since moving here?”. After, other probing questions were employed to delve into the research issue.

In the interviews with formal carers, we used open-ended questions such as “How are the elderly received and how do they evolve throughout institutionalization?”; “Considering their histories, what do you think about institutionalization as a response to instances of abuse?”; “After their reception, what results do you identify concerning the violence that victims suffered?”; and “Do you feel prepared to work here?”. Probing and supplementary questions were also asked. 

All interviews were audio recorded and transcribed verbatim. The interviews ranged in duration from 40 to 90 min. During or after the interviews, field notes were taken. All interviews were conducted by a researcher (first author), a clinical nurse with a master’s degree and appropriate experience in conducting a qualitative study. To ensure assistance for any emotional discomfort by the participants, the main researcher was assisted by a psychologist from the host institution’s support and supervision team. However, no need and/or request for intervention by the participants was observed.

### 2.5. Analysis 

Braun and Clarke’s six steps [37] were used to conduct a qualitative inductive thematic analysis: (1) familiarise with the dataset; (2) develop codes; (3) generate themes; (4) develop and review themes; (5) refine and name themes; and (6) write-up [37]. First, the material was organised by systematising the initial ideas using floating reading. Second, the relevant information was grouped into comprehensible codes and the initial codes were digitally generated. Third, the codes were categorised to generate themes. Fourth, themes were validated by analysing all codes and the dataset. Fifth, the subjects were developed and labelled, resulting in a thematic tree containing inferences, interpretation, and reflective analysis [37]. In the last phase, we wrote up the analysis in an organised story about the data and topic. Themes and quotes were translated into English at this stage to check for inconsistencies. Sample excerpts were numbered in brackets according to the participant’s role (Older Victim [OV] or Formal Carer [FC]) and by interviewee number.

The MaxQDA^®^ Software–version 2018 (VERBI Software, Berlin, Germany) [38] was used to organise and manage the collected data. 

### 2.6. Study Rigour

To ensure the study’s rigour, Guba and Lincoln’s criteria of credibility, transferability, dependability, and confirmability were considered [39]. For study credibility, interaction with the participants, persistent observation, and a critical stance by the researchers (all nurses with expertise in qualitative research) were applied. Enough time was spent collecting and becoming familiar with the data. Two researchers checked and re-checked the codes to peer-check the representativeness of the data as a whole. To achieve dependability and confirmability, we used representative quotations from the transcribed text and all the authors tried to reach the best organisation of data. No major disagreements were noted, but unclear phrases and sentences were discussed and agreed upon. Moreover, any discrepancies between codes, subcategories, and categories were debated until an agreement was achieved. The study team unanimously concluded that the data were well-saturated due to the ease of data grouping (investigator triangulation and data triangulation). A thorough analytical explanation of the context, method, and limitations was introduced for transferability.

### 2.7. Ethical Considerations

The study complied with all ethical and legal issues regulated by resolutions 466/2012 and 510/2016 of the Brazilian National Health Council and based on the Helsinki Declaration. Approval was obtained from the Permanent Commission for Project Evaluation and the Permanent Committee on Ethics in Research with Human Beings (COPEP) (protocol CAAE: 08167419.7.0000.0104). Before the interview began, each participant provided written informed permission. All personal information was decoded, and each participant was given a unique number. The code list was kept in a locked file cabinet only accessible by the research team. Given the sensitivity of the study topic, participants were assured that their identities would be kept confidential. Moreover, each victim’s complete story would not be presented.

## 3. Results

### 3.1. Participants’ Characteristics

Participants included eight institutionalised older adults and ten professionals from the LSIE (Table 2). All elders were retired and living on a minimum wage. Before admission in the LSIE, only one lived in their own house, five lived alone, one lived with family members, one lived in another LSIE, and one was discharged from a psychiatric hospital. Regarding the reasons leading to institutionalisation, all participants were victims of some type of abuse, such as economic or property exploitation, physical and psychological violence, neglect, or family abandonment. The mean time of institutionalisation in the LSIE was 2.8 years. All formal carers were female, with two nursing aides, three nursing technicians, and five personal care workers. No LTC workers had received specific training in care for the older victims of abuse.

### 3.2. Findings from Thematic Analysis

**Figure 1:**
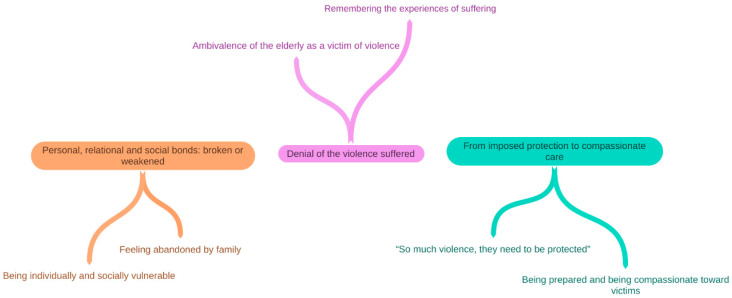
Thematic map: an overview of findings.

The qualitative analysis generated three thematic categories and six subcategories to understand the institutionalisation process of elder abuse victims: (1) personal, relational, and social bonds: broken or weakened; (2) denial of the violence suffered; and (3) from imposed protection to compassionate care. An overview of the themes and subthemes is depicted in Figure 1.

#### 3.2.1. Personal, Relational, and Social Bonds: Broken or Weakened

A combination of individual, relational, and societal factors contributes to the risk of becoming a victim of elder abuse. The lack of safe family relationships was observed as a factor that characterised the urgent need for the immediate protection of the older person.

##### Being Individually and Socially Vulnerable

The social context in which they were inserted was strongly related to triggering cases of violence and abandonment. In the documentary analysis, it was common to find medical diagnoses, such as disorders due to the use of alcohol and/or multiple drugs, or even a family history of mental illness. There were also older people with a history of aggressive behaviour, life in subhuman conditions, extreme poverty, and even homelessness, among other adverse situations.

One of the older victims whose alcohol abuse and aggressive behaviour led to marital separation (which intensified these behaviours and weakened the bonds with their children), mentioned:

*My life was different after I got separated, my life ended. My children were moving away. (...) After this happened in my life, where did I end up? (...) I feel bad for her, for my ex-wife, because I’m only here because of her. If what happened in my life hadn’t happened, I wouldn’t be here, I’d be at my house... she cheated on me because I drank*.

(OV-1)

Another victim added:

*When my son was 20, he left home, used drugs and I was sad. Today he has schizophrenia because of drugs, my wife left, and he lives in a health centre (...), now it’s up to me. When I think about my story, I realise the difficult environment I lived in*.

(OV-7)

One of the carers also highlighted the complexity of personal histories that characterise the lives of abuse victims who arrive at the unit.

*Some of the cases that arrive at the institution are situations of enormous vulnerability, most of them motivated by the weak family and social support network, in addition to extreme poverty*.

(FC-2)

The presence of mental and behavioural disorders among older adults, compounded by a low socioeconomic context, has been shown to be associated with situations of abandonment and other types of abuse.

##### Feeling Abandoned by Family

In long-stay institutions, there is a record of visits to the older person. These visits can be extemporaneous or regularly scheduled (weekly). The fragility of family ties was evidenced by the scarcity of visits received. Some seniors never received visits, others rarely received them, and only two received frequent visits. The participants’ narratives point to the fragility of these bonds:

*I found my daughter again after a year of living here at home (...) I had no contact with my family. And I know that the home nurse insisted a lot on my daughter coming to visit me. Even so, I don’t always see her*.

(OV-1)

*I feel abandoned by my family. Our relationship is not good, it’s like I don’t have brothers (...) I’ve always taken care of everyone, now they don’t even visit me*.

(OV-3)

*I didn’t have much contact with my family, sometimes I saw my brother passing by on the street because he lived close by. I just miss the people I know, who would have breakfast at my house, I am grateful for them, they were the only ones who helped me*.

(OV-8)

Positive feelings were expressed in socio-affective relationships with non-relatives, emphasising the fragility of family relationships. The fragility of bonds with relatives, even if denied by some older people, appeared between the lines, in their sad faces when talking about these relationships, suggesting they wanted more affection in family relationships.

#### 3.2.2. Denial of the Violence Suffered

The denial of the older adults regarding the family violence suffered is common and was expressed through the narratives of the victims, which contradicted the facts retrieved from each victim’s documentary files.

##### Ambivalence of the Elderly as Victims of Violence

Some older participants did not accept their status as victims, despite showing various signs of violent situations, neglect, and abandonment, which may represent, in part, a poor understanding of the phenomenon of violence and abuse. At the same time, interviewees felt a need to state that they remained autonomous in their decisions regarding living in the institution and that being admitted to the institution was just a personal decision and not a protective measure:

*I haven’t seen my children for a long time, but there was no violence! I came by my own will*.

(OV-1)

*I never experienced violence. I came to live here because I had already lived with all my brothers and it didn’t work out, we fought a lot and I thought it was better to come to live here*.

(OV-6)

Like the fragility and rupture of family ties, situations of violence, abandonment, and neglect perpetrated by family members are often not recognised by older adults. The reasons for this denial are diverse, with emphasis on feelings of shame, failure in family relationships, and rejection.

*There was no violence, my family just doesn’t remember I exist. When they brought me to live in the home, I lived alone. My sister kept my bank card and I had nothing at home. The home staff already knew me and brought me food*.

(OV-3)

*There was no problem in the family. I feel sorry for my son and my sister, because, when I needed it most, they referred me to other people to take care of me*.

(OV-7)

Violence against older people is still considered a strictly family matter, diluting itself in the domestic sphere, as a reserved and intimate context.

*My daughter sometimes offended me… I didn’t like it, but I never dared to talk about it with anyone… it was a family matter, but it reached a limit*.

(OV-2)

##### Remembering the Experiences of Suffering

The interviews, the document analysis, and the researcher’s perceptions recorded through field notes indicate that the elderly—even when aware of their histories, relationships, and conflicting family contexts—acted as if they were ashamed and blocked their experiences and memories of the difficult times they lived through. They refused to speak or justified their silence [about the violence suffered] by resorting to professional guidelines, contradicting their reports.

*There are things in life that are not good to remember... and the doctor asked me to practice this because it doesn’t matter anymore*.

OV-4)

*(...) I was afraid of being alone, on the other hand, I felt like a burden to them*.

(OV-6)

At times when emotion was evident, the older participants tried to disguise and divert the subject, thereby exposing the suffering of remembering their stories.

#### 3.2.3. From Imposed Protection to Compassionate Care

Faced with the complexity of situations of violence, it is imperative to protect victims. In this regard, carers are sensitive to the pain of elders, which generates satisfaction through compassion.

##### “So Much Violence, They Need to Be Protected”

The types of violence suffered by the older participants were physical, psychological, financial/patrimonial, neglect, and, most commonly, abandonment. In all cases, the perpetrator was a family member. Both violence and institutionalisation are related to an increase in the older person’s dependence, exhaustion of care possibilities, lack of psychological support from the family, and the fragility of family ties.

The severity and high frequency of cases of violence against older people were recognised by carers during their activity and justified the immediate institutional care to protect these victims:

*Suffering violence caused by children is terrible, I think there is nothing worse. We have an older woman who suffered sexual violence and the perpetrator was her brother. These occurrences hurt us. We have cases of aggression, both verbal and physical, false imprisonment (...) in short, violence of all kinds, always caused by the family*.

(FC-2)

*Most of the time, families are the ones who abuse the elderly, and here we deal with all types of violence: psychological, financial, physical, and abandonment. So much violence, they need to be protected. We do everything to restore the health of the elderly and their joy of life. Most of the time, we succeed*.

(FC-3)

Some participants highlighted the degree of physical dependence, multiple health morbidities, and precarious hygiene conditions, caused or exacerbated by the violence resulting from fragile family relationships. On the other hand, in most cases, in the absence of a safe family context, institutionalisation became the only means of immediate protection.

*The older people we welcome arrive very weak, sick, scared, and in subhuman hygiene conditions. Everyone needed to be institutionalised because no other previous assistance was provided (...) so it got to the point it was*.

(FC-1)

*I ate what I could cook (...) I walked with difficulty and the doctor said that I could no longer live alone, because of my health, but I had nowhere to go, I paid rent and the son of the owner of the house I rented, he helped me*.

(OV-5)

Another important finding refers to the scarcity of family support responses, community intervention in terms of prevention, and health and social assistance policies. Institutionalisation needs to be the last alternative and not the first option. The older people suffer during adaptation and there are extremely necessary rules to guarantee collective rights, good coexistence, and excellence in care. The professionals’ narratives clarified:

*I think that in some cases something else could have been done, it could have been useful to help the family (...) the professionals could help with the care of the elderly at home, without having to separate them from the home environment. After suffering violence, it is necessary to take them away from the family. Some cases that are here in the hospital are those who were in a situation of abandonment l, and really needed to be institutionalised to be able to live with dignity*.

(FC-1)

*I understand that other measures could be taken before the admission, such as helping with care at home and close to the family or even bringing the family closer together before definitive separation, as they suffer a lot in the adaptation period. Institutionalisation is full of prejudice, especially by the elderly*.

(FC-2)

Transitioning to an LSIE is a particular type of change, when the older person experiences a discontinuity in their life that can lead to dissatisfaction and maladjustment.

##### Being Prepared and Being Compassionate toward Victims

The LSEI’s welcoming environment has transformed victims’ lives. Professionals recognised that they have the skills to care for victims of violence. However, this knowledge was most often acquired empirically (based on the experiences in the unit and exchanging experiences with peers), since they were not required to have experience or prior specialised geriatric care training at the time of hiring.

*I realise that the management of the secretariat [of the Ministry of Health] sometimes does not understand the complexity of these services. Our training is every day, it is part of the work routine. We make our observations and discuss the cases together, in this way, the care improves*.

(FC-2)

The severity of cases of violence received at the institution awakens empathic understanding and compassion in carers, reflecting positively on the relational dimension of care activities, and on the centrality of care for the older people and their circumstances.

*The work is not easy, but I notice that the team is very engaged and cares with love, everyone does a little more (...) The cases are so serious that many are referred for immediate care by the office for the elderly*.

(FC-4)

*Most servers relate well with the elderly, treat them with affection, talk, and have patience. (…) I’m enjoying it, I feel good. I like to take care of the elderly*.

(FC-3)

The staff was satisfied with themselves for rising above the everyday obstacles, taking ownership of their responsibilities, and persisting. Their shared preparation serves as a source of inspiration for LTC workers to remain committed to their professional role in assisting victims.

## 4. Discussion

Our findings summarise how the institutionalisation of older victims of violence takes place given the fragility of family ties. In addition to the biological aspect, the elderly’s family and affective relationships are important and can impact the life course of this population [1]. Human development is a cyclical and ecological process that is not restricted to an initial period of life [26] but continues with the ageing process, generating results from the interaction between constitutive systems and environments. Therefore, the transition between each of the environments involved and the established affective quality influence the developmental trajectory [18].

The way families are organised affects their relationships and the systems in which they are inserted. The findings showed that there are direct links between the fragility of family relationships and the urgent situations that motivate institutional care and possible family reintegration.

Periodic visits to institutionalised older people by their family members are important to maintain or rebuild bonds and should be encouraged [22]. However, the reality is that intervals between visits become longer and longer, even if the institution is aware of and encourages family relationships. Often, the family bonds are only motivated by previous family dependency [20,23].

From an ecological perspective, the study indicates that relationships are a source of support in situations of change and crisis and, conversely, their absence or poor quality has a negative impact. Among the victim’s relational factors, social isolation and reliance are risk factors highly connected with abuse [39].

The older person’s context of vulnerability—due to social, educational, or economic issues or even access to healthcare—can promote abandonment [14,40,41]. Vulnerability tends to trigger other types of violence, increasing the possibility of the elderly person’s institutionalisation. When an older person remains under the care of their family members in a context of violence, this culminates in the violation of their rights. Therefore, institutionalisation is justified as an immediate protection mechanism for older people, given their imminent risk [18,19]. Their protection should therefore be a key policy priority. Furthermore, for older people who rely on others to ensure that they receive the best care in institutional settings, it is essential that trust and expectations in these settings not be broken [42]. Orfila et al. [39] state that institutional and informal support lowers the likelihood of mistreatment.

Despite continuous exposure to violence perpetrated by family members, many older people deny what they are experiencing. Such denial is a mechanism of self-affirming success in family relationships, entailing control over one’s history, even if it is untrue [43]. Denial also reveals ignorance of the types of violence against older people that may be caused by the family, a very common fact in today’s society. Denying the perpetrator’s accountability implies “that the mistreatment situation will be perceived as less serious when it is attributed to contextual circumstances seen as extrinsic to the perpetrator that are amenable to stress or burden, particularly if the victim internalises a sense of blame for these circumstances (e.g., requiring care from the perpetrator, shared living with the perpetrator, financial strain)” [44] (p. 882).

For older people who suffer from abuse, recalling memories can trigger negative feelings. The literature suggests that remembering intrusive memories in the aftermath of trauma might result from an impaired ability to forget disturbing material [45,46]. In addition, the expression of their ideas may be linked to a past of varied, inert feelings, which directly influence the speeches when instigated to remember.

Following frequent exposure to violence and subsequent institutionalisation, older people gradually move away from their family life and, sometimes, trigger a process of loss of autonomy [47]. Autonomy is a basic value for older people. Thus, violence might undermine the abused person’s autonomy in three senses: “(1) it threatens the abused person’s survival and safety; (2) it focuses the abused person’s attention on the interests of the abuser, preventing her from pursuing the basic human goals of survival, safety, self-actualization, and wellbeing; and (3) it submits the abused person to the will of the abuser, causing her to prioritize his goals above her own basic human needs” [47] (p. 141). 

Increased dependency also increased the risk of being subjected to paternalistic behaviour. This sort of ageism is generally created by common paternalistic perceptions of older people as weak, frail, helpless, and in need of protection by a younger, stronger society. But such protection, albeit well-meaning, can constrain older people’s autonomy [48]. In contrast, studies have indicated that older people who live in the community have a higher chance of making autonomous decisions about their health care than those who are institutionalised [49]. Older people feel that expressing their opinions and maintaining their rights enhances their autonomy. Upon receiving institutional protection, the victim assumes that institutionalisation was a choice and not the result of fragility in their social relationships and development throughout life [14,50]. LTC workers can promote or hinder older people’s autonomy in a variety of ways, such as offering chances for autonomy, managing daily care requirements and activities, and limiting older people’s choices [51]. Although not mentioned by our participants, evidence indicates that strict daily institutional routines can decrease the elderly’s control over their lives and their decision-making ability [51].

In Brazil, according to the current legislation, institutionalisation is the last instance of integral protection for older people whose rights were violated [18,20,23,50]. Before institutionalisation, there is an assistance policy that manages the support, guidance, and follow-up for older people in conflicting situations. First, all possibilities to guarantee an effective family life for the older person and re-establish their relationships must be exhausted. These actions must assume the strengthening and recovery of family and community ties before institutionalisation or the construction of new references, when applicable [20,23,50].

Once welcomed into an LSIE, elder abuse victims are under the care of professionals. This is a vast field of action, production of knowledge and power, full of singularities that require expertise and competence on the part of the professionals who practice institutional care [15,40]. The physical–structural and organisational aspects of long-stay institutions must also comply with rules and regulations. Thus, there is an urgent need for cultural adaptation and training of professionals to adequately serve this population [13,15], with care centred on the person and their circumstances [5,22].

In Brazil, demands for institutional protection come especially from social assistance services, health policies, police stations, and/or the Public Prosecutor’s Office. Failures in the flow of primary social assistance occur because, after identifying contexts of family vulnerability, the necessary interventions to prevent and protect against violence are not always implemented. Referrals to LSIEs should occur only after interventions in a natural context (community context) have been determined unsuccessful. According to the evidence, this should constitute a last option, because, in an attempt to eliminate the violence, it often generates a rupture between the elderly and their family. However, LSIEs need to be considered as a means of immediate protection, namely in cases of high severity, dependency, caregiver burden, and the breaking of family and social ties [20,40,50].

Our findings corroborate the need for attention to life histories and the reasons for institutionalisation, in addition to medical diagnoses and the care itself, for which, formal documentation is vital [15,26]. The lives of the elderly and their stories, after being embraced, are transformed and in constant movement, requiring they be listened to and closely monitored [18,19].

### 4.1. Study Limitations

As a qualitative study, our findings should be understood in the local setting where the data were collected. The decision to recruit participants from an institutional setting provided the participants with a safe atmosphere for the interviews, whereas their home or other contexts might have been less safe or more readily controlled by an abuser. Despite this study’s strength, we were limited to a single institution with a small sample size; and our findings were influenced by the cultural and regional characteristics of the participants. Therefore, cross-cultural studies in different settings involving other conditions of abuse are recommended. Recruitment for the interviews was dependent on institutional arrangements, which limited our ability to achieve a meaningful mix of participants. Despite our efforts to recruit as diverse a sample as possible to obtain a comprehensive view, the heterogeneity was limited. Our study was further constrained to a single interview per participant, implying we had no opportunity to validate findings. Lastly, we did not separate the perspectives of victims from those of formal carers during thematic analysis but treated them as a whole. Nonetheless, the study may serve as a springboard for additional theoretically driven studies on this topic, notably in healthcare. Future studies should involve collaboration among experts from various disciplinary backgrounds.

### 4.2. Implications for Practice

Based on the findings, we highlight the need to design or adopt strategies that respect the older person’s individuality, their life context, as well as their expectations and projects. From a preventive standpoint, communities can create structures to meet the specific needs that guarantee the right to age with dignity and security, that is, to be part of a greater social project in which everyone should be included, including older people. On the other hand, there is an emerging need to raise awareness and re-educate societies regarding elder abuse [52]. To this end, we suggest the creation of specific training actions and preventive social programs in schools and public and private institutions, to deconstruct prevailing ageist stereotypes still culturally present in today’s society. The media, advertising, and art can play an essential educational role in combating ageism.

In parallel, measures to protect older victims of abuse should include, first, a specific legal framework to effectively protect the elderly, based on prevalence studies and special attention to indicators of financial violence and neglect (which can take on different and unforeseen forms). Second, we must reach a consensus on the definition of abuse against older people and its typologies, respecting the culture of each country. In this sense and aiming to protect all elderly Brazilians, we suggest the implementation of a Commission for the Protection of the Elderly at a national level, with its own competencies for the protection of older people in danger [53]. The existing support structures were created solely for intervention at the local level, and their regulatory management is clearly insufficient and must be updated, since it does not cover the entire national territory, causing discrimination among the elderly and, consequently, social inequality. However, measures should not rely solely on public policies and compliance with laws that value older people. We must urgently build a collective conscience that encourages society to search for improvements and train skilled professionals that respond to the real needs of a largely unprotected and endangered older generation [54], preventing the progressive worsening of the situations revealed by the current ageing population. Lastly, we need to create more easily navigable response systems with multi-professional teams capable of responding to the complex demands of elder abuse victims [55]. Likewise, educational measures for LTC workers are needed to promote person-centred care and equip them with skills to handle elder abuse cases [54].

## 5. Conclusions

Our findings reflect how ruptures in socio-affective relationships can be a potential aggravating factor for the different types of violence in old age and, therefore, precipitate the institutionalisation of older adults. The fragility of older people’s family ties, so often ignored, has a negative impact on a personal and social level, accelerating the need for institutionalisation.

These aspects underline the importance of developing public policies aimed at preventing violence against older people and strengthening affective bonds, especially family ties. There is a need for effective services that guarantee that families have the conditions to care for their elderly without losing access to work, income, education, and health, preventing the removal of older people from their natural context, and consequently avoiding institutionalisation. In Brazil, institutionalisation is considered the last measure, with priority given to family and community insertion. Thus, long-term care services for older people must focus on intersectionality and interdisciplinarity, in order to guarantee support for the needs of older people and their support networks.

## Figures and Tables

**Table 1:** Inclusion and exclusion criteria for participants.

	Inclusion Criteria	Exclusion Criteria
Elders	Adults ≥ 60 years (United Nations [35] defines older persons as those aged 60 years or over) Admitted to the institution due to family abuse Willing to be interviewed	Cognitive impairment (evaluated by Mini-Mental State Examination [36]) that prevents participation Hospitalised during the data collection period
LTC Workers	Provide direct assistance to older adults Integrated in the institution for more than six months	Absent from work activities due to vacation or sick leave.

**Table 2:** Characteristics of participants.

Variables	Elders	LTC Workers
**Age (years) mean (SD; range)**	74.25 ± 7.22 (60–85)	48.1 ± 10.65 (31–63)
**Sex**		
Male	4	0
Female	4	10
**Years of schooling**		
<8 years	7	0
≥8 years	1	10
**Income**		
<1 minimum wage ^†^	8	0
≥1 minimum wage	0	10
**Time in LSIE**		
<1 year 1–5 years	2 5	0 6
6–10 years 11–15 years	1 0	3 0
≥16 years	0	1

^†^ In 2023, the minimum wage in Brazil was R$1302.00 per month. SD = standard deviation.

## Data Availability

This paper is a part of the master’s dissertation of the first author, and all data generated or analysed during this study are included in this article.
